# High impact of bacterial predation on cyanobacteria in soil biocrusts

**DOI:** 10.1038/s41467-022-32427-5

**Published:** 2022-08-17

**Authors:** Julie Bethany, Shannon Lynn Johnson, Ferran Garcia-Pichel

**Affiliations:** 1 School of Life Sciences, Arizona State University, Tempe, AZ 85287 USA; 2 Center for Fundamental and Applied Microbiomics, Biodesign Institute, Arizona State University, Tempe, AZ 85287 USA; 3 Los Alamos National Lab, Los Alamos, NM USA

**Keywords:** Soil microbiology, Microbial ecology, Cellular microbiology

## Abstract

Diverse bacteria lead a life as pathogens or predators of other bacteria in many environments. However, their impact on emerging ecological processes in natural settings remains to be assessed. Here we describe a novel type of obligate, intracellular predatory bacterium of widespread distribution that preys on soil cyanobacteria in biocrusts. The predator, *Candidatus* Cyanoraptor togatus, causes localized, cm-sized epidemics that are visible to the naked eye, obliterates cyanobacterial net primary productivity, and severely impacts crucial biocrust properties like nitrogen cycling, dust trapping and moisture retention. The combined effects of high localized morbidity and areal incidence result in decreases approaching 10% of biocrust productivity at the ecosystem scale. Our findings show that bacterial predation can be an important loss factor shaping not only the structure but also the function of microbial communities.

## Abstract (web summary)

Some bacteria act as pathogens or predators of other bacteria, but their impact in natural settings is often unclear. Here, Bethany et al. describe a new type of obligate, intracellular predatory bacterium of widespread distribution that preys on soil cyanobacteria in biocrusts and thus severely impacts biocrust productivity.

## Introduction

Biological soil crusts (biocrusts) are topsoil microbial communities driven by photosynthetic organisms that typically develop in ecosystems with sparse higher plant cover, notably in aridlands^1,2^. There, they provide significant ecosystem services ranging from carbon^3^ and nitrogen fixation^4^, to soil resistance to erosion^5^, dust entrapment^5^, modification of soil surface temperature^6^ and hydrology^7^. Globally, biocrusts contribute sizably to biogeochemical cycles: 15% of terrestrial net primary productivity and nearly 50% of biological nitrogen fixation^8^.

For over seventy-five years^9,10^, many bacteria that lead a life as predators of other bacteria have been described^11^. These “predatory prokaryotes” evolved independently within several bacterial phyla and are reported from many microbiomes including those of oceans^12^ and freshwaters^13,14^, sediments^11^, soils^15,16^, sewage^14^, man-made systems^17,18^, and host-associated gut microbiomes^19,20^; they clearly fill a recurrent and widespread ecological niche. And yet, their impact on emerging ecological processes in natural settings remains to be evaluated. We have recently documented the devastating impact of an unknown prokaryotic disease agent, a predatory bacterium, during the production of biocrust cyanobacterial inoculum intended for arid land soil restoration^18^.

Here we trace the finding of catastrophic failure of biocrust production in artificial settings to the presence of a naturally occurring, novel obligatory predatory bacterium that preys on dominant, non-heterocystous filamentous biocrust-forming cyanobacteria, and that we describe as *Candidatus* ‘Cyanoraptor togatus’. Unrelated to other known predatory prokaryotes, it has the typical life cycle of an endobiotic predator, with both intracellular cell division and extracellular attack stages, and the unusual trait of being an ambush predator. We further show that it, or closely related organisms, are globally distributed in biocrusts. Importantly, its activity causes significant reductions in biocrust functionality (net primary productivity, moisture retention and dust entrainment as well as shifts in carbon and nitrogen pools) locally, approaching a net effect at the ecosystem scale of 10% depression of primary productivity.

## Results and discussion

### Tracing the symptomology of predation through macroscopic plaques

**Fig. 1:**
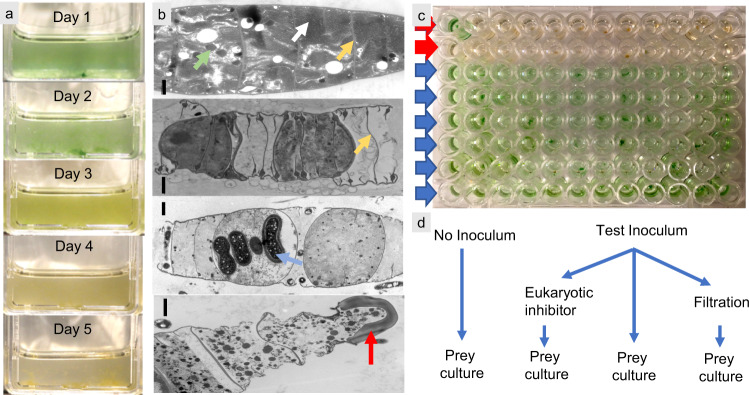
EMMA bioassay (Expanded *Microcoleus* Mortality Assay), used to study biocrust pathogens. **a** Typical visual progression of a positive EMMA inoculated with soil or culture to be tested, as used to test for pathogenicity to *Microcoleus vaginatus* PPC 9802 in the field and in enrichments. **b** Typical degradation of cyanobacterial biomass during an EMMA displayed through electron microscopy: healthy *Microcoleus vaginatus* PPC 9802 filaments (top) display abundant photosynthetic membranes (white arrows), peptidoglycan cross-walls (yellow arrows) and carboxysomes (green arrow). As infection proceeds (downwards), patent degradation of intracellular structures follows, leaving only cellular ghosts in the form of peptidoglycan wall remnants (yellow arrows), including the characteristically enlarged peptidoglycan “bumper” of terminal cells (red arrow). Intracellular bacilloid bacteria can sometimes be observed (blue arrow). Cyanobacterial cultures lose all viability. Scale bars = 1 µm. *n* = 250 images from 4 independent experiments. **c** Assay modification used in flow cytometry/cell sorting, showing enrichments positive for predation in the top two rows and those negative for predation below. **d** Test and controls in EMMA to ensure prokaryotic nature of the disease agent.

**Fig. 2:**
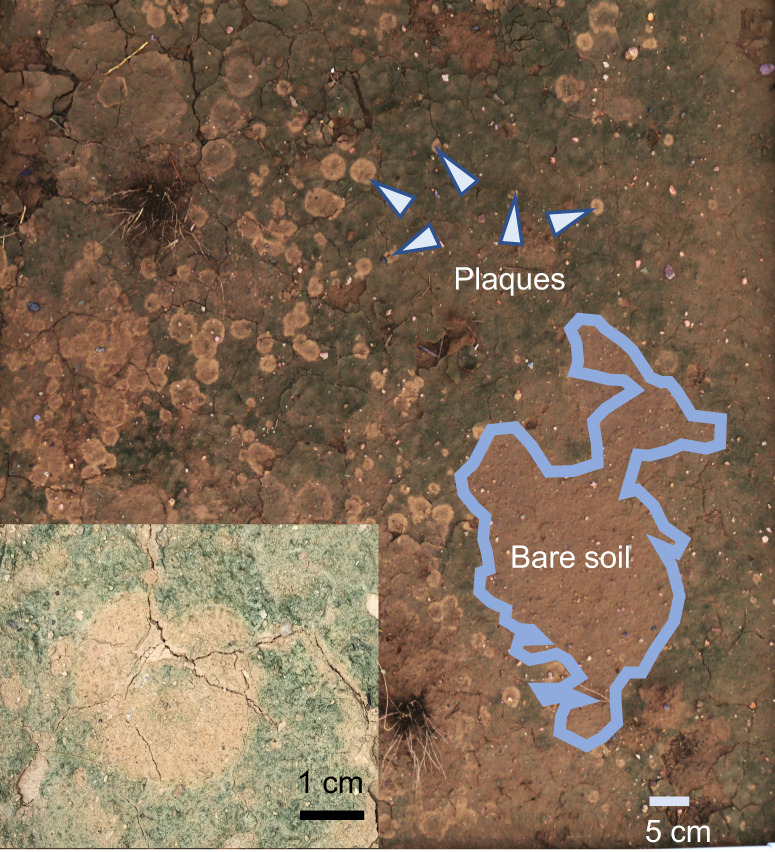
Symptomology in nature: biocrust plaques. Main: Macroscopic view of a soil surface colonized by cyanobacterial biocrusts and impacted by multiple plaques as taken after a rain in a quadrat used for field surveys. Insert: Close-up of a single plaque, showing well-demarcated boundaries and a typical central area of new cyanobacterial colonization.

A culture bioassay (Expanded *Microcoleus* Mortality Assay, or EMMA) (Fig. 1 and see Materials and Methods) based on the capacity of a soil to induce complete mortality in the foundational biocrust cyanobacterium *Microcoleus vaginatus* helped us trace the pathogen detected in biocrust production facilities to the development of cm-sized plaques, or zones of cyanobacterial clearing, in natural biocrusts. These plaques were revealed to the naked eye (Fig. 2) when the soil was wet (i.e., after a rain event), as impacted areas would fail to green up by the migration of cyanobacteria to the surface^21^, enabling us to detect and quantify them with relative ease. Soil samples obtained from such plaques (*n* = 30) from different sites (*n* = 6; Table S1) in the US Southwest were invariably EMMA + , and the pathogens always filterable with pore sizes 0.45–1 µm but not larger, and always insensitive to the eukaryotic inhibitor cycloheximide, indicating the agent’s prokaryotic nature and small size, while paired samples from asymptomatic areas just outside the plaques were always EMMA- (Table S2). These end-point EMMA solutions never gave rise to cyanobacterial re-growth upon further incubation and maintained its infectivity of fresh cyanobacterial cultures for up to 6 months. A one-time, small-scale sampling across a plaque at intervals of 2 mm using microcoring^22^ showed that the boundary of the visible plaque demarcated exactly the end of infectivity, samples 0–2 mm outside the plaque proving non-infective. Further, inoculation of healthy, natural biocrusts with EMMA + suspensions resulted in the local development of biocrust plaques, and soil from these plaques was itself EMMA + , in partial fulfillment of Koch’s postulates. Yet, standard microbiological plating failed to yield any isolates that were EMMA + (we tested 30 unique isolates), even though standard plating with similar isolation efforts can successfully cultivate a large portion of heterotrophs from biocrusts^23^.

### Cultivation, identification, and salient genomic traits of the cyanobacterial pathogen

**Fig. 3:**
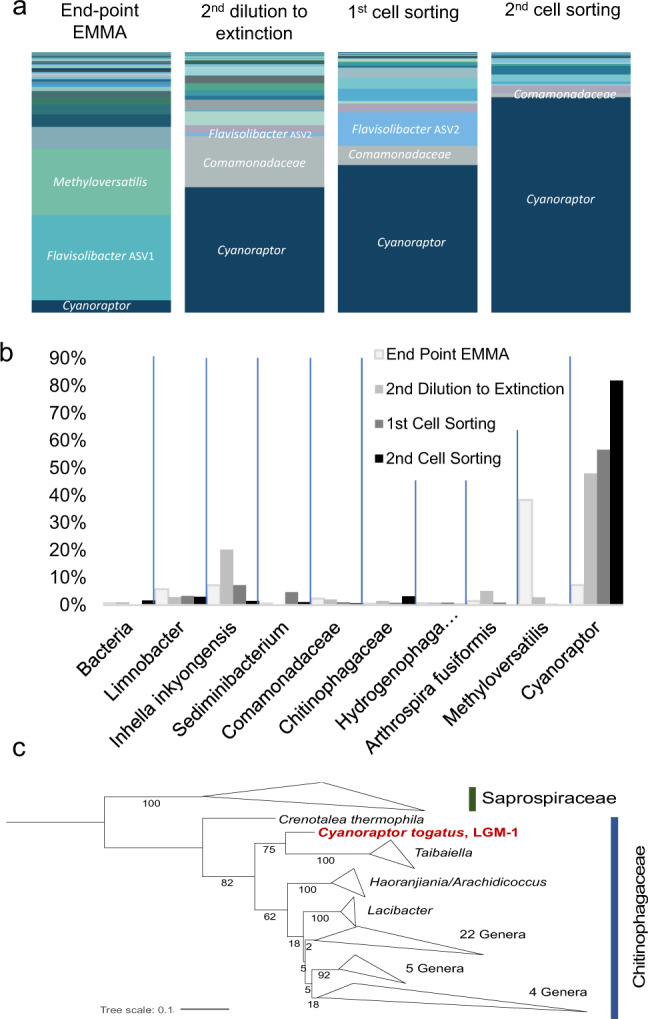
Identification of the predator. **a** Community composition of bacteria in the progressive enrichment of predator/prey co-cultures after different approaches, as labeled, based on analyses of 16S rRNA amplicons, excluding *M. vaginatus*. Two ASVs were detected for the genus *Flavisolibacter*. EMMA denotes the Expanded *Microcoleus* Mortality Assay. **b** Detailed dynamics of relative abundance for the 10 ASVs (amplicon sequence variants) detected at each stage. ASVs are labelled according to the finest taxonomic assignment possible. Each color represents an ASV, labeled at the genus label, when possible. **c** Phylogenetic relationships of the full 16S rRNA gene sequence of Cyanoraptor togatus LGM-1 obtained from its full genome sequence, against those of all existing isolates in the family Chitinophagaceae, using those in the family Saprospiraceae as an outgroup. The tree encompasses 181 individual sequences, collapsed for clarity; percent bootstrap values are at the nodes. Source data are provided as a Source Data file.

To study these organisms, we turned to enrichment of pathogen/prey co-cultures based on repeated passages through EMMA and differential size filtration combined with dilution-to-extinction approaches, followed by purification with flow cytometry/cell sorting. The process was monitored by 16S rRNA gene amplicon sequencing, and eventually yielded a highly enriched co-culture of the cyanobacterium with a genetically homogenous (one single Amplicon Sequence Variant) population that made up more than 80% of reads (Fig. 3 a, b). We name the organism represented by this ASV *Candidatus* Cyanoraptor togatus. That it corresponds indeed to the predator is supported by the fact that of the 17 ASV’s detected in the final enrichment, only 10 were consistently detected at all infectious stages in the process and, among these, only our candidate ASV steadily increased in relative abundance through the enrichment process (Fig. 3 a, b). This final enrichment of *C. togatus*, LGM-1, constitutes the basis for downstream biological and molecular analyses. Its ASV was most similar to little-known members of the family Chitinophagaceae in the phylum Bacteroidetes. LGM-1’s genome was sequenced and assembled into a single 3.3 Mb contig with 1,781 putative and 1,328 hypothetical genes (Table S3), though most proteins had low identity (<70%) to their homologs in *Chitinophaga pinensis*, the nearest relative with a fully sequenced genome, although not particularly closely related to it. The 16S rRNA gene sequence from LGM-1’s two identical copies, was only 90% similar to that of its closest isolate in culture according to BLAST (version BLAST + 2.11.0) searches. This sequence was used to narrow LGM-1’s phylogenetic placement (Fig. 3c) indicating its affinity with members of the Chitinophagaceae, but basally so, and distinct enough to make a definite assignment at the family level uncertain. Notably, no other reported predatory prokaryotes are related to LGM-1, although at least in one case, a fungal endosymbiont has been reported that shows phylogenetic affinities to the genus *Chitinophaga*^24^. LGM-1 represents yet a new branch for the notoriously polyphyletic guild of predatory prokaryotes. Some genomic comparisons to other predatory bacteria can be made (Table S3). Unlike obligate symbionts, predatory bacteria undergo no reduction in genome size^25^, possibly due to their reliance on an extracellular stage. Cyanoraptor’s genome size, if somewhat smaller, is not atypical of that of members of the Chitinophagaceae, which cluster around 4.5 Mb. Many bacterial predators lack complete amino acid biosynthetic pathways^26^. Similarly, Cyanoraptor only has full biosynthetic pathways for glutamine and asparagine. This, together with our inability to obtain prey-independent cultures, suggests that Cyanoraptor is obligately predatory. Further, predators tend to contain a wide breadth of hydrolytic enzymes and no quorum sensing genes^26^. Fitting this pattern, 3% of Cyanoraptor’s genes were assignable to hydrolases, and it also lacked quorum sensing genes. Finally, a common genomic trait of predatory bacteria is the presence of the mevalonate pathway for isoprenoid biosynthesis, which is rarely found in bacteria, but common in Eukaryotes. It has been suggested that predators are able to scavenge acetyl-coA, the initial molecule in the pathway, rather than using the energy expensive process to synthesize it from pyruvate and G3P. However, Cyanoraptor deviates from this and only contains the common bacterial pathway. Cyanoraptor is apparently non-motile in any of its life stages, lacking visible flagella, and lacking genes ascribable by similarity to known motility functions (flagellar or gliding) or taxes in its genome. This is unlike all other known predatory prokaryotes.

### Molecular detection and abundance of C. togatus

**Fig. 4:**
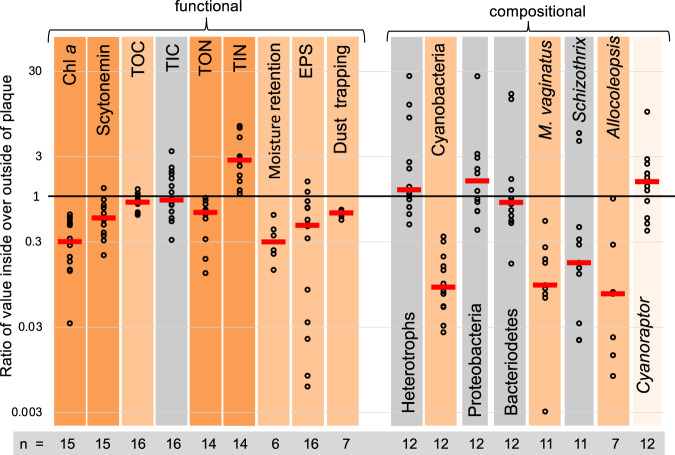
Compiled paired ratios of functional parameters and compositional (relative) abundance in biocrusts across plaque boundaries (circles), red bars denoting the medians for each group of ratios, and bar background color denoting the *p*-values that the median is significantly different from unity (Wilcoxon paired ratio two-sided tests), where gray is non-significant (*p* >  0.1), light orange is 0.05  > *p* *<* 0.1, medium orange is 0.05  >  *p* *<* 0.005 and dark orange is *p*  <  0.005. Exact *p*-values can be found in Supplementary Tables 4, 8, 9 and 10. TOC, TIC, TON and TIN stand for total organic carbon, total inorganic carbon, total organic nitrogen and total inorganic nitrogen, respectively. EPS stands for exopolysaccharide. *n* = number of biologically independent paired samples; absolute values are provided in Tables S4, S8–S10 and raw data are provided as a Source Data file.

LGM-1’s 16S rRNA sequences also allowed us to probe biocrust communities for its presence in plaques and healthy crusts surrounding them. *Candidatus* Cyanoraptor togatus or ASVs closely allied to it, were found on all plaques tested (*n* = 15 from 7 different sites), although we could also detect it molecularly in apparently healthy areas surrounding them (*n* = 12, from 4 sites tested; Table S4), albeit in significantly lower proportions (Fig. 4). Given the lack of infectivity in such areas, as discussed above, we must ascribe this detection to the presence of Cyanoraptor*’s* relic DNA, a phenomenon that is typical of soil environments^27^. Overall, however, Cyanoraptor-like ASVs were never dominant (<3.6% of reads; Table S5), even within plaques. Similarly, we could detect the presence of Cyanoraptor-like sequences in archived data from all available molecular surveys of biocrusts in many geographical areas beyond our physical survey (Table S6), indicating that its incidence is probably global. In these, Cyanoraptor-like sequences were even rarer, perhaps expectedly, because the datasets were not designed to capture plaques, but rather to survey biocrust diversity at large.

### Life cycle and ultrastructural features

**Fig. 5:**
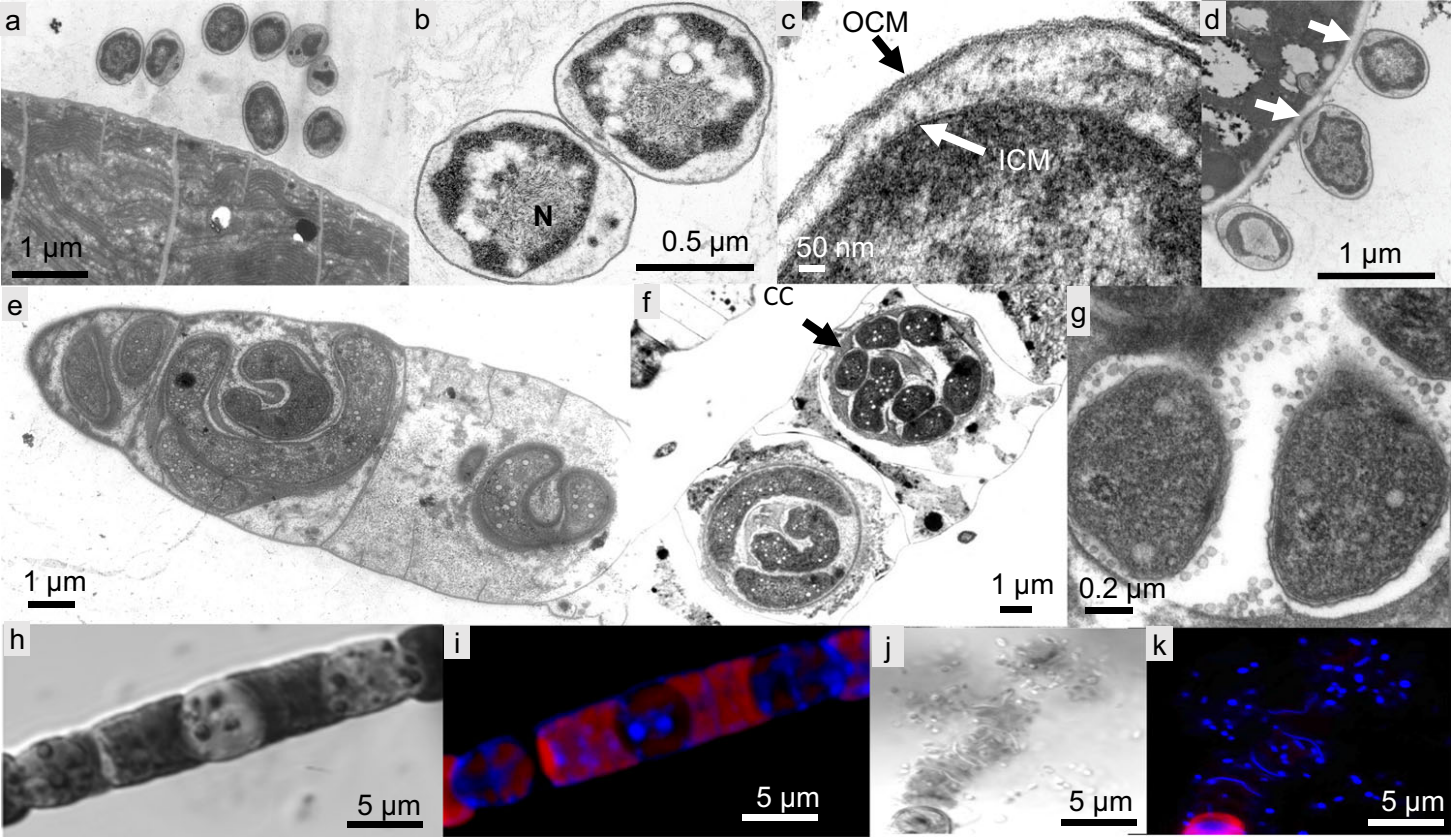
Aspects of Cyanoraptor’s life cycle through microscopy of LGM-1 and *M. vaginatus* during infection in co-culture. **a** Group of internally compartmentalized extracellular propagules close to a filament of healthy *Microcoleus*. Propagules were found in high numbers in end point Expanded *Microcoleus* Mortality Assays. **b** Close up of the ultrastructure of extracellular propagules, with inner compartment containing a nucleoid (N; Fig. S1) and electron light outer compartment or toga. **c** Detail of their Gram-negative type inner compartment (ICM) and outer compartment (OCM) membranes. **d** Docking of propagules to prey tegument, suggestive of specialized structures (arrows). **e** Non-compartmentalized, intracyanobacterial, pseudofilamentous cells (see also Fig. 6). **f** Simultaneous, multiple divisions of the Cyanoraptor pseudofilament (upper infection) and formation of an encasing, fibrilous cocoon (CC; see also Fig. 7), with another infecting cell at the pseudofilament stage (lower infection), and generalized degradation of cyanobacterial structures (see also Fig. 1). **g** Detail of the release of extracellular vesicles in late intracellular phase into the space between cell and cocoon (see also Fig. 8). **h**, **i** Optical and confocal paired images of a filament of *M. vaginatus*, with infections showing DAPI-stained Cyanoraptor and loss of photosynthetic pigment autofluorescence in infected cells. **j**, **k** Optical and confocal paired images of a fully degraded cyanobacterial filament, having released groups of extracellular propagules. **a**–**g** and **h**–**k** were selected from observations of 250 micrographs from 4 different complete experiments and 28 images from 6 different complete experiments, respectively.

**Fig. 6:**
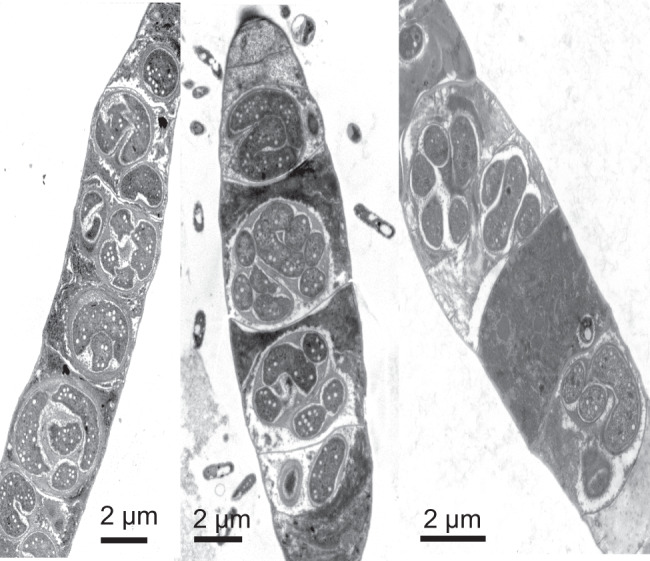
The pseudofilamentous nature of growth during early intra-cyanobacterial phase of Cyanoraptor from EM analyses. We show three selected examples (*n* = observations of 250 micrographs from 4 different complete experiments.).

**Fig. 7:**
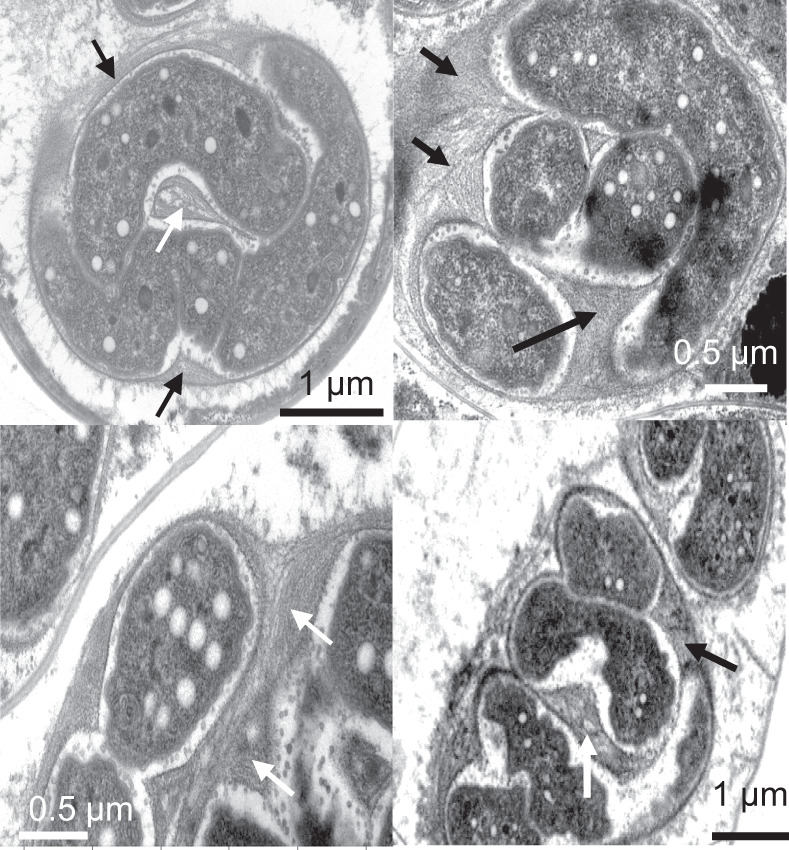
Aspects of the fibrillar cocoon typical of Cyanoraptor’s late intracellular stage as seen in EM preparations. Four selected examples are shown with arrows pointing to cocoons (*n* = observations of 250 micrographs from 4 different complete experiments.).

**Fig. 8:**
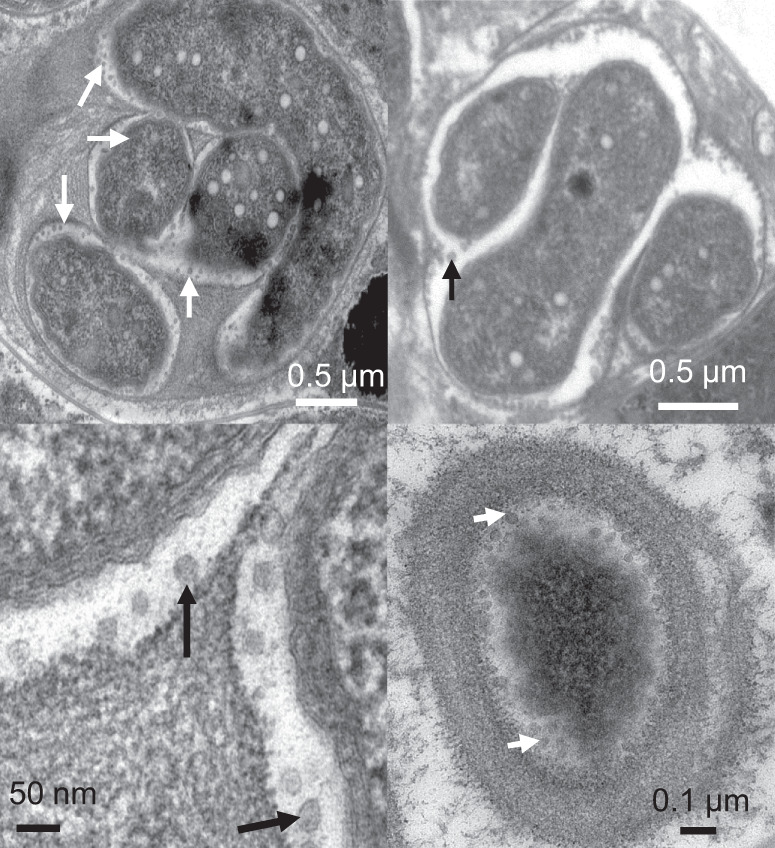
Details of the production of extracellular vesicles by late-phase intracyanobacterial Cyanoraptor as seen through EM. Four exemplary photomicrographs are shown, with arrows pointing to vesicles (*n* = observations of 250 micrographs from 4 different complete experiments.).

We studied Cyanoraptor’s life cycle by following the infection dynamics within typical EMMA through transmission electron and confocal microscopy. End-point EMMAs invariably revealed the absence of healthy filaments of *Microcoleus* (only carcass-like ghost filaments lacking cytoplasm could be seen; Fig. 1b), and a large number of small (0.8–1 µm diam), clearly internally compartmentalized, non-flagellated, Gram-negative cocci (Fig. 5a–d), which we interpret as Cyanoraptor’s extracellular propagules because of their (i) small size, (ii) high numbers and (iii) proximity to dead cyanobacteria. No dividing cells were detected among these propagules (*n* = 250; surveyed from TEM photographs). The inner, electron-dense compartment of the propagules contained a typical fibrillar nucleoid (Fig. 5b, Fig. S1) and was circumscribed by two membranes separated by an electron transparent region, as is typical of a gram-negative tegument (Fig. 5c). Two-membrane internal compartmentalization in bacteria was known previously only in the planctomycete *Gemmata obscuriglobus*^28^. The outer compartment, or “toga”, 0.1–0.4 µm thick, was electron-light and separated from the extracellular space also by two membranes with a clear interspace (Fig. 5c). Upon infection of fresh prey, the cocci in close proximity to *M. vaginatus* developed tegumentary structures reminiscent of docking zones (Fig. 5d), and as infection proceeded, predatory cells gained entry into the cyanobacterial cytoplasm. Once inside, they lost all trace of compartmentalization, starting to grow into pseudo-filamentous forms (Figs. 5e and 6), with large numbers of cytoplasmatic inclusions, as *M. vaginatus* cells were degraded showing patent loss of macromolecular structures typical of healthy cells like thylakoids and carboxysomes^29^, bulging cellular teguments due to loss of cell wall strength, and loss of cytoplasmatic contents (Fig. 5e, h). The damage, and the infection, often spread to multiple cells adjacent to the entry cell in the cyanobacterial filament (Fig. 5h). Multiple cell division occurred during Cyanoraptor’s intracellular phase only at the late pseudo-filamentous stage (Fig. 5f), accompanied by a loss of reserve polymers, the formation of a cocoon of fibrillar nature around it (Figs. 5f and 7), and by the excretion of large numbers of 10–20 nm sized membrane-bound extracellular vesicles (Figs. 5g and 8). After full degradation of *M. vaginatus* cells, propagules were released (Fig. 5j, k), remaining held in groups. We posit that the outer compartment acts as a repository for hydrolytic enzymes destined for the prey, a common strategy in predatory bacteria^30^; that most of LGM-1’s genes (3%) annotated as polymer hydrolases are endowed with signal peptides for excretion supports this contention. This outer compartment seems to be formed by fusion of the extracellular vesicles trapped between cells and fibrillar cocoon. Given its lack of motility, it would appear that Cyanoraptor uses a strategy of ambush predation that likely relies on cyanobacterial motility for encounters, docking, and possibly dispersal. This is consistent with the prey range established in the lab, where all cyanobacterial strains sensitive to it are motile by gliding (Table S7). Thus, Cyanoraptor appears to be an obligatory, endocellular predatory bacterium, the first of this type as a predator of cyanobacteria, although a variety of Proteobacteria, Bacteroidetes and Firmicutes can lyse cyanobacterial cells extracellularly^31^.

### Prey specificity in culture and in the field

Out of 70 cyanobacterial strains (in 14 genera) tested using EMMA but substituting the prey, only 14 (in 4 genera) were susceptible to attack (Table S7), all filamentous and non-heterocystous, and typical pioneer biocrusts formers. The common denominator of susceptible strains was their ability to form crowded, large bundles of filaments and to move by gliding motility, consistent with basic epidemiological principles, since this will promote prey mortality and contagion, respectively^32^. Comparisons of 16S rRNA-based community composition complemented with absolute quantification via quantitative PCR in paired samples (inside vs. outside of plaques), showed that field epidemics involved a significant population decline (*p* = 0.002; Wilcoxon paired ratio test; Fig. 4, Table S4) in cyanobacteria. While there was a tendency to cause absolute increases in other bacteria (i.e., non-cyanobacteria) this was not statistically significant (Fig. 4). No other single bacterial phylum showed significant absolute benefits or declines from the epidemic. Among the 29 cyanobacterial taxa identified in our field dataset, only *Microcoleus vaginatus*, *Allocoleopsis sp*., *Potamolinea sp*. and *Xeronema sp*. suffered demonstrably significant and consistent loses (*p* < 0.05; Table S8), in line with the prey range demonstrated in culture. The affinity of Cyanoraptor for *M. vaginatus*, likely the most abundant terrestrial cyanobacterium^33^, and to some of members of the Coleofasciculaceae, of wide distribution across continents^34^, affords it a veritable worldwide buffet.

### Functional consequences of *C. togatus* epidemics

**Fig. 9:**
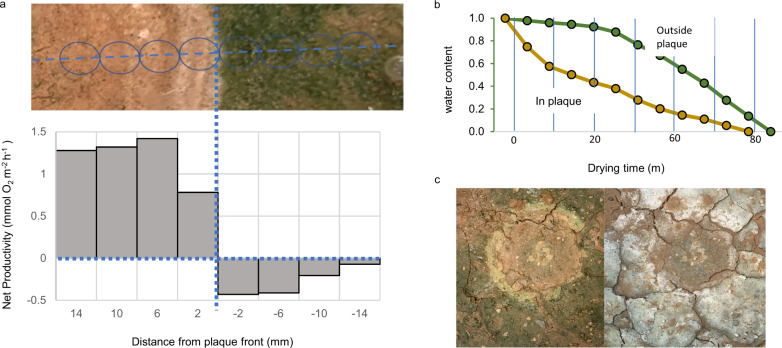
Functional consequences of Cyanoraptor epidemics as derived from paired analyses of parameters inside vs. outside plaques. **a** Typical distribution of net primary productivity across a plaque boundary (top photo). **b** Exemplary dynamics of soil desiccation inside vs. outside of a plaque. **c** Example of differential dust trapping within and around a plaque before (left) and after dust application. Results for replicate experiments are in Table S9. Source data are provided as a Source Data file.

The ecological consequences of Cyanoraptor epidemics were assessed by comparing how relevant biocrust properties were affected across plaque boundaries. A result compilation of the tests carried out is in Fig. 4 and the full sets of data and statistics are in Tables S9 and S10. The most severe effect was on net primary productivity, which was invariably and fully obliterated in crossing from healthy biocrusts into plaques, turning them into net respiratory systems, the inflexion point coinciding spatially with the plaque boundary (Fig. 9a, b). Functional effects on oxygenic photosynthesis were much more severe than one could have surmised from losses of cyanobacterial 16S rRNA genes, again indicating the likelihood of relic cyanobacterial DNA blurring the full extent of morbidity by Cyanoraptor. Total Organic Carbon (TOC) and Total Organic Nitrogen (TON) content were also consistently lowered (by 13% and 38%, in average, respectively), concurrently increasing soil levels of Dissolved Inorganic Nitrogen (DIN) by 300% in average, which in absolute terms was roughly commensurate with TON loses (Table S10). The biocrust content of extracellular polysaccharide (EPS), responsible for some of the hydrological and dust-trapping character of biocrusts^35^, was also consistently reduced in plaques (by 53% in average), possibly as a consequence of the release of DIN, which would render EPS a better substrate for growth for heterotrophs when N is available. Not surprisingly, important functional properties of biocrusts like moisture retention capacity during desiccation, and dust trapping ability were also negatively impacted (Figs. 4, 9c), by 67 and 36% respectively. It can perhaps be surprising that a bacterium that is relatively rare can elicit such devastating, cascading effects. However, Cyanoraptor’s feeding seems to be quite inefficient, destroying much more than it can reap, and making cyanobacterial biomass available to a range of other adventitious bacteria. A rough estimate of this biomass transfer efficiency from microscopic images points to values well below 1% (Table S11).

### Scaling impacts to the ecosystem level

**Fig. 10:**
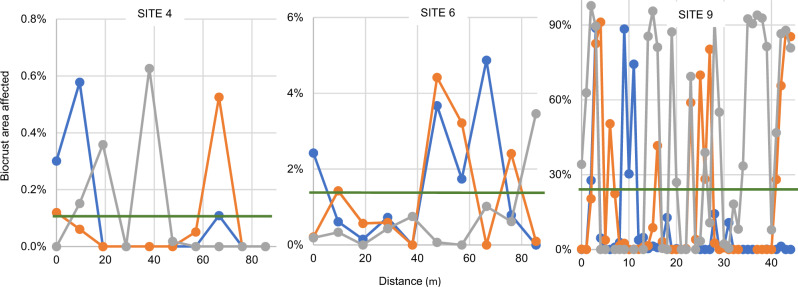
Landscape-scale surveys of plaque incidence at three sites used to assess ecosystem impact of the disease. Each color denotes a separate transect, and the site means are the green horizontal lines. Full dataset in Supplementary Data 1.

All the effects discussed above pertain to concentrated epidemics that span scales of cm. However, scaling such effects to the ecosystem level requires an assessment of the incidence and distribution of individual plaques at much larger scales. While plaques were found in all sites inspected, to scale-up more quantitatively, we carried out photographic field surveys of the incidence of plaques larger than 3 mm in 195, 1-m^2^ quadrats along 9 linear transects at 3 geographical locations, chosen for their high biocrust cover and carried out during rainy days to make plaques conspicuous (Fig. 10, Supplementary Data 1). Plaque density in single transects ranged from 1 to 23 m^−2^, averaging 9.0 ± 8.6 m^−2^, and affecting 8.3 ± 14.3% of the biocrust area surveyed. Single quadrats ranged from 0 to 263 plaques m^−2^ and from 0 to 98% areal infection. Plaques strongly followed aggregated distributions across scales (Fig. 10), even within quadrats (Nearest Neighbor test; *p* = 0.01). Scaling single-plaque functional effects on an area basis by simple arithmetic, it follows that the ecosystem-level consequences of Cyanoraptor infections are also significant, tithing primary productivity in the order of 10%. This must be considered an underestimate, as only fully formed plaques larger than 3 mm were counted, and Cyanoraptor was detected molecularly also in areas without conspicuous plaques.

### Bacterial predation as loss factor and consequences for soil restoration

Since losses to viral infection or protistan/meiofaunal grazing remain to be quantified in biocrusts, prokaryotic predation stands, for now, as the single most important biological loss factor in these microbiomes. In the light of our work, prokaryotic predation should be considered, along with other biological loss factors, as a potentially important determinant of population dynamics in microbiomes. Further, biocrust restoration practices that are based on using whole communities for production of inoculum run the risk of spreading infection and should be performed with caution. Since our original report of catastrophic failures due to cyanobacterial mortality^18^, we have been able to successfully avoid disease spread by simply testing for diagnostic plaques in the starting material, and discarding any containing plaques in the downstream production.

### Description of *Candidatus* Cyanoraptor togatus, *genus novum et species nova*

*Candidatus* Cyanoraptor, gen. nov., Cy.a.no.rap’.tor, Latinized Gr. m.n. Cyanos, blue-green and L. raptor m.n, plunderer, M.L. Cyanoraptor m. n a plunderer of the blue-green (cyanobacteria).

Prey-dependent intracellular Gram-negative predatory bacteria in the Bacteroidetes with affinities to the family Chitinophagaceae, showing conspicuous cell differentiation into distinct intra- and extracellular stages in its life cycle. Extracellular propagules are non-dividing, coccoid, internally compartmentalized by a double membrane. The inner compartment holds the nucleoid. Intracellular stages lack an outer compartment, grow into rods and eventually pseudo-filaments, to undergo simultaneous, multiple cell division. No motile phases exist. Flagella are absent. Likely auxotrophic for many amino acids.

*Candidatus* Cyanoraptor togatus. sp. nova. To.ga´.tus, L. *togatus* m. adj., dressed in a robe or toga, in reference to the outer compartment in the propagules.

Extracellular cocci 0.93 ± 0.15 µm, intracellular cells 1.02 ± 0.41 µm wide and more than 5 µm long. Preys on non-heterocystous, motile filamentous terrestrial cyanobacteria. Strain LGM-1^T^, in enrichment form, and its genome, are the type material. Its genome is 3.3 Mb with a 42% G + C content. Isolated from biological soil crust in Arizona, USA. Maintained as a co-culture with *M. vaginatus* PCC 9802 as prey.

## Methods

### Sampling and surveying

We sampled wet cyanobacterial biocrusts from seven different sites in the Sonoran and Chihuahuan deserts using inverted 10 cm Petri dishes, letting them dry out in the open before storage for downstream processing. The sites used for sampling can be found in Table S1. Some of these sites were chosen for intensive spatial surveys because of the presence of widespread biocrust cover. We conducted 9 linear transects, 3 in each site, always after significant rains to make plaques easily visible. Transects were 45 m long with quadrats 1 m apart in Site 9, and 85 m with quadrats 8.5 m apart elsewhere. Quadrats were documented photographically (Fig. 2), photographs then analyzed using ImageJ Fiji^36^. Derived parameters obtained were (a) the percent of biocrusts area impacted by plaques, (b) the areal density of plaques and c) the distance from a plaque to its nearest neighbor to characterize spatial plaque distribution.

### Expanded *Microcoleus* Mortality Assay (EMMA)

To determine the presence of prokaryotic agents pathogenic to cyanobacteria we developed a bioassay based on the fate of an axenic culture of *Microcoleus vaginatus* (PCC 9802). 10 ml of Jaworski’s minimal liquid medium in 75 mL vented-cap tissue culture flasks was inoculated with PCC 9802 (0.2 mg (Chl *a*) l^−1^) as prey, and with 25 mg from a homogenate of the soil to be tested, incubated under 10 µmol m^−2^s^−1^ of illumination with a 12 h photoperiod. The assay invariably included a) uninoculated negative controls, and b) negative controls in which the soil was autoclaved before assay inoculation. All assays and controls were run (at least) in triplicate and simultaneously. Flasks were read after 5 days of incubation by simple inspection, positives showing patent chlorosis first and full degradation of cyanobacterial biomass (Fig. 1a). If positives were found, two more tests were done on PCC 9802 prepared as above: (1) 1 mL of positive end-point suspension was inoculated into cultures and the eukaryotic inhibitor, cycloheximide, added to a final 12.5 µg/ml, positives indicating a non-eukaryotic pathogen, and (2) aliquots of the end-point suspension were added to fresh cultures after filtration through polycarbonate filters with nominal pore diameters of 0.2, 0.45, 0.8 and 1.0 µm. Negatives at or below 0.45 µm indicated a non-viral agent.

### Enrichment cultivation of Cyanoraptor

We carried out a sequential battery of enrichment/isolation techniques, starting with direct plating of size-filtered contents of positive EMMA assays on tryptic soil broth solidified with 1.5% agarose, but none of the resulting isolates was EMMA + . For enrichments, we first used size-fractionation (SF) of positive EMMA end-point aliquots to enrich for bacteria 0.2 µm−1 µm in nominal size, that were then inoculated on *M. vaginatus* (PCC 9802) cultures, with several filtration passages. A dilution-to-extinction (DTE) in 1/10 steps to 10^−7^ based on the SF preparations, with ten replicates for dilution, followed. The highest dilution that still killed *M. vaginatus*, was then used for recurrent rounds of DTE. Yet, SF/DTE did not produce a pure co-culture of predator and prey. Flow cytometry/cell sorting (FCCS) was subsequently used to further purify the enrichment. SF/DTE preparations were filtered (<1 µm) and injected into a BD FACSAria Ilu cell sorter. Cells between 0.79 µm and 1.3 µm in diameter were sorted into one hundred 96-well plates containing healthy, axenic *M. vaginatus* (PCC 9802) cultures and incubated under standard MMA conditions (Fig. 1c). This FCCS procedure was performed twice in sequence. The final enrichment stage was used for downstream analyses under strain denomination LGM-1.

### 16S rRNA gene community analyses

DNA from cultures or field samples was extracted via the Qiagen DNeasy PowerSoil Kit (QIAGEN, catalog number 12888-50), following manufacturer’s instructions. The V4 region of the 16S rRNA gene was amplified using primers 515 F and 806R^37^. PCR reaction, amplificate quantification, and Illumina sequencing have been published^18^. The raw FASTQ files were de-multiplexed within the MiSeq Illumina workflow under default parameters. Paired sequences were de-multiplexed and analyzed via Qiime2 2019.7^38^, using the DADA2 plugin to create a feature table with representative sequences (ASVs) and their frequency of occurrence. Taxonomy was assigned with the Naive Bayes classifier trained on the Greengenes 13.8 release. Cyanobacterial (=oxyphotobacterial^39^) sequences phylogenetically assigned separately using Cydrasil^40^. To assess and quantify the presence of Cyanoraptor-like sequences in field or database datasets, matches were sought against the full 16S rRNA sequence of *Candidatus* C. togatus by BLAST.

### Genome sequencing

DNA from LGM-1 was extracted using the Monarch Genomic DNA Purification Kit (Thermo Fisher, catalog number K0512) and sheared with G-tube (Covaris) at 4000 rpm using manufacturer’s suggested methods. Following bead cleanup, DNA ranging in size from approximately 8 to 15 kb was used to construct the Pacbio sequencing library (10 kb) was constructed using the Pacbio Express II protocol and reagents (SMRTbell Express, catalog number NC1811322). Sequencing was performed on a Pacbio Sequl II instrument (Pacbio) following manufacturer’s protocols, with the following parameters: binding kit 2.0, primer V4, sequencing plate 2.0, 8 M v2 cell, loading concentration of 55 pM, sequencing time of 30 hours with CCS mode, yielding a FASTQ file. Following raw sequencing data collection, CCS analysis was performed using default settings on SMRT Link V8, with an average quality score of QV41. The raw FASTQ file was imported into a local instance of EDGE (Empowering the Development of Genomics Expertise)^41^ for contig assembly using 10% of data. Assembly was performed with Irasm-wtdbg2 into 1416 contigs at 22.97-fold coverage. Contig binning, identification and quality checks used CheckM^42^. The genome of the predatory bacterium was identified using the existing partial 16S rRNA gene information and the resulting (single) bin was then curated with SEED on the RAST-Server (Rapid Annotation using Subsystems Technology)^43^. Initial annotations were performed using Prokka^44^ with transfers from the closest relative, *Chitinophaga pinensis*. All putative genes, not hypothetical genes, were then cross-referenced using UniProt (Universal Protein Resource)^45^. Proteins of interest were then confirmed using pBLAST on the NCBI database. The KEGG database (Kyoto Encyclopedia of Genes and Genomes)^46^ was used to detect the presence of metabolic pathways of interest with further manual annotations.

### Phylogenetic placement

A reference tree using the maximum-likelihood + thorough bootstrap (1000 replicates) method and the GTRGAMMA model from the 145 16S rRNA sequences (1485 + bases) publicly available from cultured members of the Chitinophagaceae and 36 sequences from cultured members of the Saprospiraceae as an outgroup, after alignment with SSU-ALIGN. Poorly aligned columns were removed based on a 95% confidence profile. Tree topology was inferred on the CIPRES^47^ high-performance computing cluster, using the RAxML-HPC2 workflow on XSEDE. The resulting tree was imported and visualized on the iTOL^48^ 3 server.

### Microscopy

For TEM, enrichments of the strain LGM-1, were used to follow two individual infection cycles with daily subsampling over the course of 5 days. Subsamples were pelleted and fixed with 2.5% glutaraldehyde and sodium cacodylate wash buffer (0.1 M, pH 7.4) at room temperature for 2 h and then suspended in agarose, fixed with 1% buffered OsO_4_ at 4 °C for 2 h and block stained in 0.5% uranyl acetate at 4 °C, overnight, dehydrated with increasing concentrations of acetone and rinsed twice with 100% propylene oxide at room temperature. Pellets were then infiltrated, at room temperature with rotation, with successive levels (10%, 25%, 75%, 100%) of epoxy resin for 1, 2, 8, and 12 h, respectively. Infiltrated pellets were embedded in fresh 100% resin and held at 60 °C for 24 h. Pellets in resin were thin sectioned (70 nm) and post-stained with 2.5% uranyl acetate/lead citrate. Sections were imaged using a JEOL 1200EX TEM and a Philips CM 12 TEM at 80 kV. Preparations of strain LGM-1 were also observed during an infection cycle by confocal fluorescence and DIC microscopy after fixation with 2.5% glutaraldehyde and staining with 0.1 mg/mL DAPI (4’−6-diamidino-2-phenylindole) on a Zeiss SM 880 microscope. DAPI stained LGM-1 cells were observed with excitation at 358 nm and emission at 461 nm. *M. vaginatus* was observed by its autofluorescence based on photosynthetic pigments (emission at 663 nm with excitation between 620 and 630 nm).

### Cyanobacterial pigments

Chlorophyll *a* and scytonemin areal concentrations were used as proxies for cyanobacterial biomass and heterocystous cyanobacteria biomass in biocrusts. Three mm diameter, 1 cm deep cores were taken, extracted in acetone and their pigment concentration determined spectrophotometrically following the methods of Giraldo-Silva^49^.

### C and N content

Total organic nitrogen (TON), total organic carbon (TOC), total inorganic nitrogen (TIN) and total inorganic carbon (TIC) content were determined within a plaque and in healthy biocrust within 5 cm from the edge of the plaque, from cores with depth and diameter of 0.5 cm. Cores were ground to a fine powder in a SPEX Certiprep 8000D mill and milled for 5 minutes. For TOC/TIC we used the acid fumigation on a Perkin Elmer series II CHNS/O analyzer. For TON and TIN content, milled cores as above were used for the potassium chloride (KCl) extraction of nitrate and ammonium or for total nitrogen (TN) on a combustion analyzer.

### Extracellular polysaccharide content (EPS)

EPS was extracted with the EDTA method^50^ from 1 cm deep, 1 cm wide cores and then quantified using the phenol-sulfuric acid method^51^ with a commercial kit from Sigma-Aldrich (catalog number MAK104).

### Net primary productivity/net respiration

Primary productivity was assessed using oxygen exchange rate determinations over the crust surface^22^. For this we used benthic flux chambers^52^, miniaturized to a measuring area of 12.6 mm^2^ (4 mm diameter circular opening) to a total volume of 26 µL. This miniaturization ensured internal mixing by convection. The chambers were provided with an internal O_2_-measuring microoptode (50 µm) tip diameter, connected to a Fire-Sting O_2_ oxygen meter, both from Pyroscience Gmbh. Chambers were set in place over the crust using a micromanipulator. The optode was calibrated at 100% saturation, and temperature corrected in real time (19–20 °C). For incubations, biocrust held in 15 cm Petri dish bottoms were wetted to saturation with deionized water, let stand at 20 µmol photons m^−2^s^−1^ of illumination for 4 hours, then placed inside a larger glass circular container (17 cm diam. and 6.5 cm tall), submerged in deionized water to about 2.5 mm above the surface of the crust, under an air-current driven flow and illuminated at saturating 350 µmol m^−2^s^−1^ of white light (Fiber-Lite Illuminator model 190), as measured with a quantum meter (Li-Cor model LI-250). The benthic chamber was then brought down onto the desired area to seal with the crust surface using the micromanipulator. Measurements at each spot were carried out in triplicate. The O_2_ exchange rates were back calculated, using the measuring area, the chamber capacity and an O_2_ saturation of 8.79 mg/L (19.5 °C, 0% salinity, 335 m elevation). By convention, exchange rates bear a positive sign when they signify a net export of O_2_ from the crust whereas O_2_ consumption rates bear a negative sign.

### Desiccation dynamics

Desiccation dynamics of biocrusts were followed using two temperature-corrected commercial (UP Umweltanalytische Produkte GmbH) conductivity-based mini-probe according to ref. 53, placed to a depth of 2 mm, either inside or just outside plaques. Starting from saturated soil, desiccation was sped up by a fan overhead, and measurements were taken concurrently every 10 min. Dynamics showed typically a delay in desiccation outside the crusts, and hence we report this delay as the time needed to reach a water content of 80% saturation.

### Differential dust trapping and binding assay

Biocrust samples were wetted with distilled water, photographed from above, and placed inside of a larger container, which was filled with a 50 g L^−1^ suspension of diatomaceous earth to a level 4-5 cm above the crust surface, and let stand for several hours for particles to settle. The sample was carefully removed and let stand subaerially under a gentle stream of air until the surface was completely dry. The dry diatomaceous earth layer imparted a strong surface reflectance to the crust. The sample was placed again in the large dish, which was then filled with distilled water to about 1 cm above the crust and subjected a stream of air to scour surface particles. The sample was retrieved from the dish, placed under a stream of air to slowly dry out, and photographed sequentially to “develop” qualitatively spatial patterns of differential trapping. For quantification, we used RGB image analyses (Fiji) of areas of interest (either plaques or healthy biocrust around them) using the blue channel, which gave us the least divergence among different bare soils. The average pixel intensity was calculated for a given area after the test and normalized by the average pixel intensity of the same area before the test. The normalized average pixel intensities of plaque over biocrusts areas, pairwise, were obtained as a measure of differential dust trapping.

### Statistics

To test for statistical significance in general patterns between parameters inside and outside of a plaque, ratios of the paired values (inside vs. outside) in each plaque were calculated, and the probability that the median of the collection of ratios was significantly different from unity assessed with a paired-sample Wilcoxon test for ratios^54^. Additionally, differences in means by site, when needed, were assessed using Welch’s t-tests. For microbial community analyses, significance in composition shifts at the ASV (amplicon sequence variant) level for heterotrophic bacteria and species level for cyanobacteria were also tested with pairwise PERMANOVAs calculated on Bray Curtis similarity matrices of relative abundances derived from sequencing with 9999 permutations. To determine potential drivers of community composition shifts at the ASV level, the maaslin2 package^55^ was used. All calculations were performed using R^56^.

### Reporting summary

Further information on experimental design is available in the Nature Research Reporting Summary linked to this paper.

## Supplementary information

Supplementary Information

Description of Additional Supplementary Files

Reporting summary

Supplementary Data 1

## Data Availability

The sequencing data generated in this study have been deposited in the NCBI database under BioProject PRJNA786587, BioProject PRJNA730549 and BioProject PRJNA730811. All other data generated in this study are provided in the Supplementary Information. Source data are provided with this paper.

## Source data

Source Data
