# Supplementary material for: High impact of bacterial predation on cyanobacteria in soil biocrusts
Source: Nat Commun. 2022 Aug 17;13:4835. doi: 10.1038/s41467-022-32427-5 (PMC9385608; doi:10.1038/s41467-022-32427-5)
Supplement: Supplementary file 1 — Supplementary Information [file 41467_2022_32427_MOESM1_ESM.pdf]

High impact of bacterial predation on cyanobacteria in soil biocrusts

Supplementary Tables

Supplementary Table 1. Spatial survey of incidence and distribution of Cyanoraptor plaques in transects of three sites in the southwestern US.

Sites	Coordinates	Type of crust*	Closest City, State	Desert Biome
1	lat 32.59194°, long -106.85286°	Smooth	Las Cruces, NM	Chihuahuan
2	lat 32.50321°, long -106.74097°	Smooth	Las Cruces, NM	Chihuahuan
3	lat 32.56348°, long -106.75795°	Smooth	Las Cruces, NM	Chihuahuan
4	lat 32.51540°, long -106.74269°	Rugose	Las Cruces, NM	Chihuahuan
5	lat 34.33703°, long -106.72910°	Rugose	Albuquerque, NM	Chihuahuan
6	lat 32.9913°, long -111.76130°	Smooth	Casa Grande, AZ	Sonoran
7	lat 33.3923°, long -111.35404°	Smooth	Gold Canyon, AZ	Sonoran
8	lat 33.57293°, long -111.79713°	Rugose	Scottsdale, AZ	Sonoran
9	lat 33.30089°, long -111.68285°	Smooth	Mesa, AZ	Sonoran

* Type of crust as defined in Belnap and Lange 2001[80].

Supplementary Table 2. Correlation between plaque symptomology and the presence of pathogenic bacteria. For each location samples were tested inside and outside of plaques using EMMA, Expanded *Microcoleus* Mortality Assay.

Desert Biome	Site*	Number of positives: Inside	Number of positives: Outside	Replicate plaques
Sonoran	Site 7	6	0	n=6
Sonoran	Site 6	6	0	n=6
Chihuahuan	Site 1	6	0	n=6
Chihuahuan	Site 2	6	0	n=6
Chihuahuan	Site 5	6	0	n=6

*Site Information according to Table S1

Supplementary Table 3. Summary of LGM-1's genomic properties.

General		
	Genome Size	3.3 Mbp
	Number of putative genes	1,781
	Number of hypothetica genes	1,328
Similarities with predatory bacteria		
	Lack of quorum sensing genes	No matches found for *lasM, rhlR, vsmR*, ahl
	Lack of complete amino acid biosynthesis pathways	Alanine, arginine, aspartic acid, cysteine, glutamic acid, glycine, histidine, isoleucine, leucine, lysine, methionine, phenylalanine, proline, serine, threonine, tryptophan, tyrosine, and valine missing most enzymes in pathway
	Diverse suite of genes for hydolases	4 cell wall amidases, 1 glycosidase, 44 cell wall peptidases, 48 hydrolases
Differences with predatory bacteria		
	Contains the DXP isoprenoid biosynthesis pathway	Full complement
	Non motile	No matches for flagellar genes *flgB, flgC, flgE, flgF, flgG, flgK, flgL, flgF, fliG, fliM, fliN, motA, motB, flhA, flhB, fliL, fliP, fliQ, fliR, flag-1, flag-2*<br>No matches for gliding motility genes *gldA, gldB, gldD, gldF, gldG, gldI, gldJ, gldK, gldL, gldM, gldN, gldO, sprA, sprE, sprT, remA*
	Non chemotactic	No matches for chemotaxis genes *cheA, cheC, cheD, cheM, cheW*

Supplementary Table 4. Impacts of *Cyanoraptor* activity on population size of bacterial groups or taxa, as determined by 16S rRNA gene amplicon sequencing complemented with qPCR, through comparison of paired determinations (n=3 per site) inside and outside of plaques, as compiled in Fig. 4. Significance for differences in means inside vs. outside in each site are from Welch's tests. Significance for differences in the entire dataset are based on Wilcoxon tests testing that the median ratio is different from unity.

		Heterotrophic Bacteria (millions 16S rRNA gene copy number/cm^{-2})*			Cyanobacteria (millions 16S rRNA gene copy number/cm^{-2})**			Proteobacteria (millions 16S rRNA gene copy number/cm^{-2})			Bacteroidetes (millions 16S rRNA gene copy number/cm^{-2})			*Cyanoraptor*. (millions 16S rRNA gene copy number/cm^{-2})		
Site	Plaque	Out	In	Ratio	Out	In	Ratio	Out	In	Ratio	Out	In	Ratio	Out	In	Ratio
	1	33.4	115.7	3.46	85.6	56.8	0.66	8.2	50.4	6.17	4.5	22.0	4.86	2.7	5.6	2.07
	2	51.0	36.2	0.71	125.7	36.2	0.29	14.9	10.4	0.70	13.5	6.8	0.50	5.9	1.6	0.27
	3	18.3	153.7	8.41	47.0	31.2	0.66	6.8	66.9	9.88	5.2	32.9	6.33	2.4	14.7	6.07
1	4															
	5															
	6															
	Average	34.2	101.9	4.20	86.1	41.4	0.54	9.9	42.6	5.58	7.7	20.5	3.90	3.7	7.3	2.80
	SD	16.4	60.0		39.4	13.5		4.3	29.1		5.0	13.1		1.9	6.7	
	p =	0.18			0.18			0.19			0.23			0.45		
	1	147.5	111.8	0.76	101.6	23.4	0.23	49.2	50.1	1.02	19.3	13.5	0.70	4.8	3.2	0.68
	2	132.1	72.0	0.54	43.9	18.3	0.42	44.4	26.1	0.59	13.6	8.1	0.60	4.6	1.5	0.32
	3	139.9	82.1	0.59	88.4	15.6	0.18	49.5	31.4	0.63	18.7	8.9	0.48	5.3	2.2	0.41
4	4															
	5															
	Average	139.8	88.6	0.63	78.0	19.1	0.27	47.7	35.9	0.75	17.2	10.2	0.59	4.9	2.3	0.47
	SD	7.7	20.7		30.2	3.9		2.8	12.6		3.1	2.9		0.4	0.9	
	p =	0.04			0.08			0.24			0.05			0.02		
	1	129.9	85.8	0.66	129.6	12.9	0.10	45.4	26.3	0.58	23.1	10.1	0.44	14.4	6.8	0.48
	2	252.5	168.7	0.67	206.1	53.7	0.26	129.8	75.2	0.58	41.4	26.3	0.64	19.2	17.4	0.91
	3	317.2	454.7	1.43	266.7	71.0	0.27	133.9	216.4	1.62	63.3	76.7	1.21	29.9	30.4	1.02
5	4															
	5															
	Average	233.2	236.4	0.92	200.8	45.9	0.21	103.0	106.0	0.93	42.6	37.7	0.76	11.2	12.1	0.69
	SD	95.1	193.5		68.7	29.8		50.0	98.7		20.1	34.8		10.0	7.5	
	p =	0.98			0.04			0.97			0.84			0.74		
	1	92.0	32.4	0.35	127.8	13.3	0.10	48.4	14.2	0.29	18.5	8.3	0.45	3.0	1.8	0.58
	2	23.4	21.6	0.92	58.8	6.0	0.10	12.1	10.3	0.85	4.8	3.1	0.64	0.4	1.6	3.56
	3	191.3	607.1	3.17	196.5	59.2	0.30	62.2	146.4	2.35	69.2	37.1	0.54	18.0	14.3	0.79
6	4															
	5															
	Average	102.2	220.3	1.48	127.7	26.2	0.17	40.9	57.0	1.17	30.8	16.2	0.54	7.2	5.9	1.64
	SD	84.4	335.0		68.9	28.8		25.9	77.5		33.9	18.3		9.5	7.3	
	p =	0.61			0.11			0.76			0.56			0.86		
	1															
	2															
	3															
9	4															
	Average															
	SD															
	p =															
	Grand Average	127.4	161.8	1.81	123.1	33.1	0.30	50.4	60.3	2.11	24.6	21.1	1.45	9.2	8.4	1.43
	SD	92.9	181.2		68.9	21.9		42.5	62.2		21.9	20.7		9.1	9.0	
	**p =**	**0.85**			**0.002**			**0.85**			**0.25**			**0.08**		

*We assume all non-cyanobacteria are heterotrophs. **Oxyphotobacteria

Supplementary Table 5. Molecular detection of *candidatus Cyanoraptor togatus* and allied organisms, inside of plaques from different locations based on 16S rRNA similarity, with relative abundance of positive ASVs. Sequences whose closest relative was *Cyanoraptor togatus* were considered positives.

Desert	Site	Plaque	Number of ASVs	Closest ASV (% similarity to *Cyanoraptor*)	Range of ASV Match (%)	% Reads
Chihuahuan						
	1	1	23	100	100-88	1.2
		2	21	96	96-88	0.3
		3	63	96	96-88	3.4
	4	4	39	96	96-87	1
		5	33	94	94-87	0.5
		6	24	93	93-87	0.7
	5	7	68	94	94-88	0.8
		8	56	94	94-88	2.1
		9	41	94	94-88	3.6
Sonoran						
	6	10	15	94	94-87	0.2
		11	16	100	100-87	0.2
		12	14	94	94-88	1.9

*Site Information according to Table S1

Supplementary Table 6. Worldwide detection of *candidatus Cyanoraptor togatus*, or closely related bacteria, based on published 16S rRNA gene surveys of biocrusts. A positive result for the presence of *candidatus C. togatus* is roughly at the genus level (≥ 94% similarity).

Country	Desert (Reference)	Presence	% Similarity	% Reads
Australia	Pinnacles[1]	+	99	<0.01
Australia	Simpson[2]	+	98	0.03
Botswana	Kalahari[3]	+	98	0.2
China	Gurbantunggut[4]	+	94	0.4
United States	Chihuahuan			
	Las Cruces, NM[5]	+	96	<.01
	Las Cruces, NM[6]	-	-	0
	Albuquerque, NM[7]	+	99	<.01
	Colorado Plateau[8]	+	95	<.01
	Mojave[9]	+	94	<.01
Oman	Wahiba[10]	-	-	0

Supplementary Table 7. Determination of prey range of strain LGM-1 using EMMA, Expanded *Microcoleus* Mortality Assay, with PCC9802 as the positive control. All strains tested were cyanobacteria isolated from biological soil crusts and are kept in the collection of the Garcia-Pichel laboratory. Source data are provided as a Source Data file.

Family	Genus/species	Number of strains tested	Number of strains susceptible	Percent of strains susceptible
Oscillatoriaceae				
	M. vaginatus	18	10	55%
	Lyngbya sp.	1	0	0%
Schizotrichaceae				
	Schizothrix sp.	14	2	12%
Coleofasciculaceae				
(“Microcoleus steentrupii complex”)				
	Funiculus sp.	2	0	0%
	Allocoleopsis sp.	2	0	0%
	Crassifilum sp.	3	1	33%
	Parifilum sp.	1	0	0%
	Xeronema sp.	4	1	25%
Chroococcidiopsidaceae				
	Chroococcidiopsis sp.	1	0	0%
Leptolyngbyaceae				
	Leptolyngbya sp.	3	0	0%
Phormidiaceae				
	Phormidium sp.	1	0	0%
Nostocaceae				
	Nostoc sp.	8	0	0%
Scytonemataceae				
	Scytonema sp.	7	0	0%
	Tolypothrix sp.	5	0	0%

Supplementary Table 8. Impacts of *Cyanoraptor* activity on population size of selected cyanobacterial genera, as determined by 16S rRNA gene amplicon sequencing complemented with qPCR, through comparison of paired determinations (n=3 per site) inside and outside of plaques. Significance for differences in means inside vs. outside in each site are from two-sided Welch's tests. Significance for differences in the entire dataset are based on Wilcoxon tests testing that the median ratio is different from unity.

		Microcoleus vaginatus (millions 16S rRNA gene copy number/cm^{-2})			*Schizothrix* spp. (millions 16S rRNA gene copy number/cm^{-2})			*Allocoleopsis* spp. (millions 16S rRNA gene copy number/cm^{-2})			*Potamolinea* spp. (millions 16S rRNA gene copy number/cm^{-2})			*Xeronema* spp. (millions 16S rRNA gene copy number/cm^{-2})		
Site	Plaque	Out	In	Ratio	Out	In	Ratio	Out	In	Ratio	Out	In	Ratio	Out	In	Ratio
1	1	40.8	16.8	0.41	4.3	3.0	0.70	10.7	0.0	0.00	2.3	0.9	0.40	3.0	0.7	0.22
	2	56.7	7.5	0.13	1.4	8.9	6.50	15.9	0.0	0.00	4.0	3.3	0.82	4.3	0.3	0.07
	3	23.8	6.7	0.28	0.2	5.3	24.16	8.3	0.3	0.03	2.4	1.1	0.47	2.7	0.2	0.07
	4															
	5															
	6															
	Average	40.5	10.3	0.28	2.0	5.8	10.45	11.7	0.1	0.01	2.9	1.8	0.56	3.3	0.4	0.12
	SD	16.5	5.6		2.1	3.0		3.9	0.2		1.0	1.3		0.9	0.3	
	p =	0.07			0.15			0.04			0.30			0.02		
4	1	27.8	11.2	0.40	9.6	0.5	0.05	0.8	0.5	0.66	3.1	0.2	0.08	4.7	0.7	0.15
	2	9.1	5.9	0.65	2.7	1.5	0.55	0.3	0.3	1.18	0.9	0.6	0.67	3.3	0.8	0.24
	3	16.2	3.3	0.21	3.6	1.3	0.36	1.2	0.0	0.03	1.3	0.5	0.39	9.5	0.1	0.01
	4															
	5															
	Average	17.7	6.8	0.42	5.3	1.1	0.32	0.7	0.3	0.62	1.7	0.4	0.38	5.8	0.5	0.13
	SD	9.5	4.0		3.8	0.5		0.5	0.2		1.2	0.2		3.2	0.4	
	p =	0.17			0.19			0.23			0.20			0.10		
5	1	71.2	0.8	0.01	0.0	0.0		13.6	1.2	0.09	0.0	0.0		1.2	0.2	0.15
	2	69.0	33.2	0.48	10.5	5.5	0.52	11.3	1.5	0.14	0.6	1.4	2.28	3.9	0.0	0.01
	3	65.8	16.5	0.25	0.5	0.2	0.43	74.1	19.4	0.26	11.5	0.6	0.05	4.6	0.2	0.04
	4															
	5															
	Average	68.7	16.8	0.25	3.7	1.9	0.47	33.0	7.4	0.16	4.0	0.6	1.17	3.2	0.1	0.07
	SD	2.7	16.2		5.9	3.1		35.7	10.4		6.5	0.7		1.8	0.1	
	p =	0.03			0.68			0.34			0.46			0.10		
6	1	0.0	0.0		15.9	5.4	0.34	0	0		3.0	1.4	0.46	1.3	0.0	0.01
	2	0.2	0.0	0.00	19.0	2.1	0.11	0	0		5.5	0.6	0.10	1.3	0.1	0.06
	3	3.0	0.0	0.00	82.4	5.0	0.06	0	0		17.5	1.2	0.07	2.0	0.0	0.00
	4															
	5															
	Average	1.0	0.0	0.00	39.1	4.2	0.17	0.0	0.0		8.7	1.0	0.21	1.5	0.0	0.02
	SD	1.7	0.0		37.6	1.8		0.0	0.0		11.5	0.6		1.6	0.0	
	p =	0.39			0.25			NA			0.23			0.02		
9	1															
	2															
	3															
	4															
	Average															
	SD															
	p =															
	Grand Average	32.0	8.5	0.26	12.5	3.2	3.07	11.3	1.9	0.27	4.3	1.0	0.53	3.5	0.3	0.08
	SD	27.8	9.8		22.9	2.8		20.7	5.5		5.1	0.8		2.3	0.3	
	**p =**	**0.003**			**0.32**			**0.01**			**0.05**			**<0.001**		

Supplementary Table 9. Impacts of *Cyanoraptor* activity on biocrust Chlorophyll *a*, scytonemin, and EPS concentrations as well as on net primary productivity, and dust trapping capacity through comparison of paired determinations n≥3 inside and outside of plaques, as compiled in Fig. 4. Significance for differences in means inside vs. outside in each site are from Welch's tests. Significance for differences for the entire dataset are based on Wilcoxon tests, testing that the median ratio is different from unity.

		Chl a (mg/m^{-2})			Scytonemin (mg/m^{-2})			Productivity (mmol O_2*m^{-2}*h^{-1})			EPS (mg/cm^{-2})			Difference in Dust Trapping (relative units)			Moisture Retention (m until 80% water content)		
Site	Plaque	Out	In	Ratio	Out	In	Ratio	Out (avg)	In (avg)	Ratio	Out	In	Ratio	Out	In	Ratio	Out	In	Ratio
1	1	64.1	30.3	0.47	145.3	136.2	0.94	1.5 ± 0.4	-0.4 ± 0.2		0.02	0.04	1.53	1.2	0.8	0.72	60.5	37.6	0.62
	2	82.2	48.3	0.59	174.9	141.9	0.81	1.4 ± 0.4	-0.1 ± 0.0		0.04	0.05	1.16	2.1	1.3	0.60	49.5	20.9	0.42
	3	67.8	34.4	0.51	166.0	107.5	0.65	0.6 ± 0.2	-0.7 ± 0		0.07	0.00	0.03				34.6	8.3	0.24
	4										0.03	0.00	0.08						
	5																		
	6																		
	Average	71.4	37.7	0.52	162.0	128.5	0.80	1.20	-0.3		0.04	0.02	0.70	1.65	1.06	0.66	48.18	22.27	0.43
	SD	9.6	9.4		15.2	18.4		0.60	0.3		0.02	0.02		0.69	0.32		12.98	14.66	
	p =	0.01			0.07			<0.001			0.29						0.08		
4	1	95.3	52.8	0.55	489.0	279.6	0.57	0.4 ± 0.2	-0.1 ± .01		0.16	0.12	0.76	2.3	1.6	0.69	41.2	14.8	0.36
	2	114.5	72.2	0.63	898.8	321.9	0.36	0.6 ± 0.4	-0.2 ± 0.3		0.26	0.13	0.48				51.9	11.3	0.22
	3	77.4	37.2	0.48	387.2	81.2	0.21	0.4 ± 0.2	-0.1 ± 0.1		0.29	0.00	0.01						
	4										0.11	0.00	0.02						
	5																		
	Average	95.7	54.1	0.56	591.7	227.6	0.38	0.5	-0.1		0.21	0.06	0.32				46.55	13.05	0.29
	SD	18.6	17.5		270.8	128.5		0.3	0.1		0.09	0.07					7.61	2.42	
	p =	0.05			0.13			<0.001			0.04								
5	1	137.5	24.4	0.18	203.7	97.6	0.48	0.7 ± 0.2	0 ± 0										
	2	85.0	23.3	0.27	142.3	115.1	0.81	1.3 ± .3	-0.2 ± 0										
	3	89.3	17.9	0.20	162.1	207.7	1.28	0.7 ± 0	0 ± 0										
	4																		
	5																		
	Average	103.9	21.9	0.22	169.4	140.1	0.86	0.9	-0.1										
	SD	29.2	3.4		31.3	59.1		0.4	0.1										
	p =	0.04			0.50			<0.001											
6	1	49.7	14.9	0.30	85.4	46.3	0.54	0.8 ± 0.4	-0.0 ± 0.0		0.12	0.04	0.33	1.2	0.8	0.65	57.0	8.0	0.14
	2	74.2	10.2	0.14	130.2	40.6	0.31	0.9 ± 0.2	-0.4 ± 0.3		0.07	0.06	0.90	1.4	0.921	0.64			
	3	75.0	24.7	0.33	180.4	105.5	0.58	0.2 ± 0.2	-0.1 ± 0.1		0.29	0.21	0.75						
	4										0.21	0.10	0.49						
	5																		
	Average	66.3	16.6	0.26	132.0	64.1	0.48	0.7	-0.1		0.17	0.10	0.62	1.33	0.86	0.65			
	SD	14.4	7.4		47.5	35.9		0.4	0.2		0.10	0.08		0.15	0.08				
	p =	0.01			0.12			<0.001			0.33								
9	1	105.1	5.0	0.05	231.5	89.4	0.39	0.3 ± 0.2	-0.1 ± 0.1		0.65	0.36	0.55	2.0	1.1	0.54			
	2	56.8	8.0	0.14	116.6	87.7	0.75	0.4 ± 0.2	0 ± 0		0.89	0.41	0.46	1.6	1.0	0.65			
	3	69.0	9.7	0.14	146.2	82.0	0.56	0.6 ± 0.3	-0.1 ± 0.2		0.90	0.00	0.00						
	4										0.39	0.00	0.01						
	Average	77.0	7.6	0.11	164.8	86.4	0.57	0.4	-0.1		0.71	0.19	0.25	1.78	1.05	0.60			
	SD	25.1	2.4		59.7	3.9		0.3	0.1		0.24	0.22		0.29	0.04				
	p =	0.04			0.15			<0.001			0.02								
	Grand Average	82.9	27.5	0.33	244.0	129.4	0.62	0.7	-.1		0.28	0.10	0.47	1.69	1.08	0.64	49.10	16.81	0.33
	SD	30.4	19.5		211.7	84.3		0.5	0.2		0.29	0.13		0.46	0.28		9.72	11.24	
	**p =**	**<0.001**			**0.002**						**0.002**			**0.02**			**0.03**		

Supplementary Table 10. Impact of *Cyanoraptor* activity on soil nutrients, through comparison of paired determinations n≥6 inside and outside of plaques, as compiled in Fig. 4. Significance for differences in means inside vs. outside in each site are from Welch's tests. Significance for differences in the entire dataset are based on Wilcoxon tests testing that the median ratio is different from unity.

		TOC (g/kg soil)			TIC (g/kg soil)			TON (g/kg soil)			TIN (g/kg soil)		
Site	Plaque	Out	In	Ratio	Out	In	Ratio	Out	In	Ratio	Out	In	Ratio
1	1	16.9	11.4	67%	1.2	2.3	189%	0.8	0.3	32%	0.1	0.3	303%
	2	17.1	15.7	92%	0.7	2.3	346%	0.8	0.5	63%	0.1	0.2	122%
	3	28.3	18.5	65%	10.3	17.2	167%	1.6	1.2	72%	0.1	0.2	288%
	4	24.1	30.1	125%	10.8	6.1	57%	1.3	1.3	99%	0.1	0.1	181%
	5	10.6	9.2	87%	0.7	1.5	223%	0.9	0.1	13%	0.5	1.1	233%
	6	11.9	10.0	84%	2.9	0.9	32%	1.0	0.7	70%	0.3	0.4	111%
	Average	18.2	15.8	87%	4.4	5.0	169%	1.1	0.7	58%	0.2	0.4	206%
	SD	6.9	7.9		4.8	6.2		0.3	0.5		0.2	0.4	
	p =	0.60			0.85			0.14			0.31		
4	1												
	2												
	3												
	4												
	5												
	Average												
	SD												
	p =												
5	1	25.8	23.1	90%	4.20	2.20	52%	1.4	1.0	68%	0.0	0.1	265%
	2	30.4	24.5	81%	9.75	13.20	135%	1.9	1.2	64%	0.1	0.4	687%
	3	29.4	26.9	91%	10.85	9.75	90%	1.7	1.1	64%	0.1	0.3	297%
	4	26.2	27.4	105%	13.05	14.90	114%	1.7	1.5	92%	0.1	0.4	505%
	5	15.5	10.0	65%	7.35	14.85	202%	0.8	0.6	83%	0.0	0.2	652%
	Average	25.5	22.4	86%	9.0	11.0	119%	1.5	1.1	74%	0.1	0.3	481%
	SD	5.9	7.1		3.4	5.3		0.4	0.3		0.0	0.1	
	p =	0.48			0.5			0.14			0.02		
6	1	12.4	11.8	95%	2.20	1.50	68%	0.5	0.4	68%	0.1	0.1	120%
	2	9.8	6.3	64%	1.05	0.60	57%	1.2	0.7	56%	0.5	0.7	155%
	3	9.2	5.7	62%	0.60	0.50	83%	0.7	0.1	19%	0.3	1.0	279%
	4	23.7	25.0	105%	13.55	11.05	82%						
	5	7.2	8.0	111%	3.30	3.15	95%						
	Average	12.5	11.4	88%	4.1	3.4	77%	0.8	0.4	48%	0.3	0.6	185%
	SD	6.6	8.0		5.4	4.4		0.3	0.3		0.2	0.4	
	p =	0.82			0.8			0.16			0.35		
9	1												
	2												
	3												
	4												
	Average												
	SD												
	p =												
	Grand Average	18.7	16.5	87%	5.8	6.4	125%	1.2	0.8	62%	0.2	0.4	300%
	SD	8.1	8.5		4.9	6.0		0.4	0.5		0.2	0.3	
	**p =**	**0.02**			**0.59**			**<0.001**			**<0.001**		

Supplementary Table 11. Estimation of the biomass transfer efficiency from prey (filamentous cyanobacteria) to predator (*Cyanoraptor*) from TEM/Confocal imaging.

Modal Number of continuous cyanobacterial cells affected by single infection	5
Modal number of *Cyanoraptor* propagules produced in a single-point infection	6
Volume of *Cyanoraptor* propagule (V_1, µm^3)	*0.268*
Volume of *M. vaginatus* single cell (V_2, µm^3)	*75.389*
Efficiency of biomass transfer ($6V_1/5\ V_2$; %)	**0.35**

Supplementary Figures

Supplementary Fig. S1. DNA strands within the internal compartment of extracellular *Cyanoraptor*. Scale bars= 0.1 µm. (n= observations of 250 micrographs from 4 different complete experiments.)

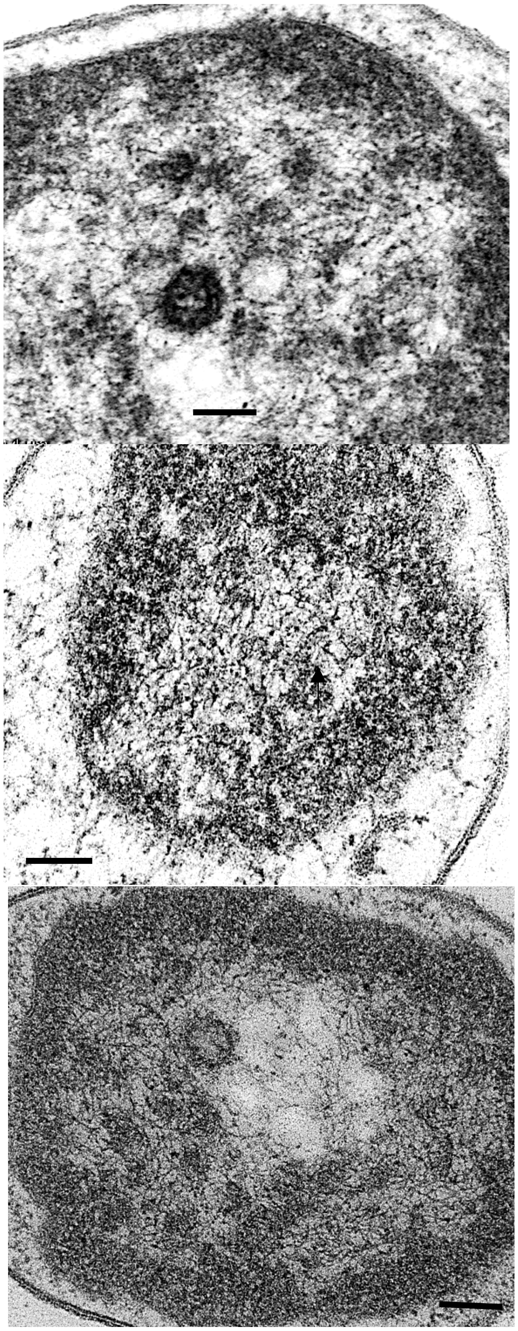

Supplementary References

1. Moreira-Grez, B. *et al.* The bacterial microbiome associated with arid biocrusts and the biogeochemical influence of biocrusts upon the underlying soil. *Front. Microbiol.* 10, (2019).

2. Elliot, D., Thomas, A., Strong, C. & Bullard, J. Surface stability in drylands is influenced by dispersal strategy of soil bacteria. *Biogeosciences* 124, 3403–3418 (2019).

3. Elliot, D., Thomas, A., Hoon, S. & Sen, R. Niche partitioning of bacterial communities in biological crusts and soils under grasses, shrubs and trees in the Kalahari. *Biodivers. Conserv.* 23, 1709–1733 (2014).

4. Zhang, B., Kong, W., Wu, N. & Zhang, Y. Bacterial diversity and community along the succession of biological soil crusts in the Gurbantunggut Desert, Northern China. *J. Basic Microbiol.* 56, 670–679 (2016).

5. Bethany, J., Giraldo-Silva, A., Nelson, C., Barger, N. N. & Garcia-Pichel, F. Optimizing production of nursery-based biological soil crusts for restoration of arid land soils. *Appl. Environ. Microbiol.* AEM.00735-19 (2019) doi:10.1128/AEM.00735-19.

6. Velasco Ayuso, S. V., Silva, A. G., Nelson, C., Barger, N. N. & Garcia-Pichel, F. Microbial nursery production of high-quality biological soil crust biomass for restoration of degraded dryland soils. *Appl. Environ. Microbiol.* **83**, 1–16 (2017).

7. Fernandes, V. M. C. *et al.* Exposure to predicted precipitation patterns decreases population size and alters community structure of cyanobacteria in biological soil crusts from the Chihuahuan Desert. *Environ. Microbiol.* **20**, 259–269 (2018).

8. Couradeau, E. *et al.* Bacteria increase arid-land soil surface temperature through the production of sunscreens. *Nat Commun* **7**, 10373 (2016).

9. Garcia-Pichel, F., Loza, V., Marusenko, Y., Mateo, P. & Potrafka, R. M. Temperature drives the continental-scale distribution of key microbes in topsoil communities. *Science.* **340**, 1574–1577 (2013).

10. Abed, R. *et al.* Habitat-dependent composition of bacterial and fungal communities in biological soil crusts from Oman. *Nat. Sci. Reports* **9**, 6468 (2019).
