# Supplementary material for: High impact of bacterial predation on cyanobacteria in soil biocrusts
Source: Nat Commun. 2022 Aug 17;13:4835. doi: 10.1038/s41467-022-32427-5 (PMC9385608; doi:10.1038/s41467-022-32427-5)
Supplement: Supplementary file 2 — Description of Additional Supplementary Files [file 41467_2022_32427_MOESM2_ESM.pdf]

Title: Supplementary Data 1.
Description: Spatial survey of incidence and distribution of Cyanoraptor plaques in transects of three sites in the southwestern US.
