# Supplementary material for: High impact of bacterial predation on cyanobacteria in soil biocrusts
Source: Nat Commun. 2022 Aug 17;13:4835. doi: 10.1038/s41467-022-32427-5 (PMC9385608; doi:10.1038/s41467-022-32427-5)
Supplement: Supplementary file 3 — Reporting summary [file 41467_2022_32427_MOESM3_ESM.pdf]

nature portfolio

Corresponding author(s): Ferran Garcia-Pichel

Last updated by author(s): May 17, 2022

# Reporting Summary

Nature Portfolio wishes to improve the reproducibility of the work that we publish. This form provides structure for consistency and transparency in reporting. For further information on Nature Portfolio policies, see our Editorial Policies and the Editorial Policy Checklist.

## Statistics

For all statistical analyses, confirm that the following items are present in the figure legend, table legend, main text, or Methods section.

| n/a | Confirmed | |
|---|---|---|
| ☐ | ☒ | The exact sample size (*n*) for each experimental group/condition, given as a discrete number and unit of measurement |
| ☐ | ☒ | A statement on whether measurements were taken from distinct samples or whether the same sample was measured repeatedly |
| ☐ | ☒ | The statistical test(s) used AND whether they are one- or two-sided<br>*Only common tests should be described solely by name; describe more complex techniques in the Methods section.* |
| ☒ | ☐ | A description of all covariates tested |
| ☒ | ☐ | A description of any assumptions or corrections, such as tests of normality and adjustment for multiple comparisons |
| ☐ | ☒ | A full description of the statistical parameters including central tendency (e.g. means) or other basic estimates (e.g. regression coefficient) AND variation (e.g. standard deviation) or associated estimates of uncertainty (e.g. confidence intervals) |
| ☐ | ☒ | For null hypothesis testing, the test statistic (e.g. *F*, *t*, *r*) with confidence intervals, effect sizes, degrees of freedom and *P* value noted<br>*Give P values as exact values whenever suitable.* |
| ☒ | ☐ | For Bayesian analysis, information on the choice of priors and Markov chain Monte Carlo settings |
| ☒ | ☐ | For hierarchical and complex designs, identification of the appropriate level for tests and full reporting of outcomes |
| ☒ | ☐ | Estimates of effect sizes (e.g. Cohen's *d*, Pearson's *r*), indicating how they were calculated |

*Our web collection on statistics for biologists contains articles on many of the points above.*

## Software and code

Policy information about availability of computer code

| | |
|---|---|
| Data collection | none |
| Data analysis | Qiime 2 used for 16S rRNA amplicon analyses. Naive Bayes Classifier (trained on Greengenes 13.8), Cydrasil 0.22a and BLAST 2.10.0 for taxonomic assignments. For genomics, EDGE (Empowering the Development of Genomics Expertise, version as of 6/2020) was used for contig binnning & identification. Quality checks were through Check M46 (version 6/2020). |

For manuscripts utilizing custom algorithms or software that are central to the research but not yet described in published literature, software must be made available to editors and reviewers. We strongly encourage code deposition in a community repository (e.g. GitHub). See the Nature Portfolio guidelines for submitting code & software for further information.

## Data

Policy information about availability of data

All manuscripts must include a data availability statement. This statement should provide the following information, where applicable:

- Accession codes, unique identifiers, or web links for publicly available datasets
- A description of any restrictions on data availability
- For clinical datasets or third party data, please ensure that the statement adheres to our policy

The sequencing data generated in this study have been deposited in the NCBI database under BioProject PRJNA786587 (https://www.ncbi.nlm.nih.gov/bioproject/PRJNA786587), BioProject PRJNA730549 (https://www.ncbi.nlm.nih.gov/bioproject/?term=PRJNA730549) and BioProject PRJNA730811 (https://www.ncbi.nlm.nih.gov/bioproject/?term=PRJNA730811). All other data generated in this study are provided in the Supplementary Information or Source Data files.

## Human research participants

Policy information about studies involving human research participants and Sex and Gender in Research.

| | |
|---|---|
| Reporting on sex and gender | NA |
| Population characteristics | NA |
| Recruitment | NA |
| Ethics oversight | NA |

Note that full information on the approval of the study protocol must also be provided in the manuscript.

# Field-specific reporting

Please select the one below that is the best fit for your research. If you are not sure, read the appropriate sections before making your selection.

☐ Life sciences ☐ Behavioural & social sciences ☒ Ecological, evolutionary & environmental sciences

For a reference copy of the document with all sections, see nature.com/documents/nr-reporting-summary-flat.pdf

# Ecological, evolutionary & environmental sciences study design

All studies must disclose on these points even when the disclosure is negative.

| | |
|---|---|
| Study description | The quantitative field component was descriptive on 3 independent sites in the US Southwest. No treatments were imposed, thus no factors or interactions are relevant. Additional sites were used for cm-sized samples destined for laboratory determinations. |
| Research sample | Ramdom samplefor lab research were taken around plaques, dried and stored dark until use. They contained the whole microbial community of interest. |
| Sampling strategy | Samples of topsoil containing plaques were taken randomly to encompass both healthy and deseases areas, determining size of 10 cm . Intervals of 1m were used for transect plots with samples 1 m2, replicate transects with 30-100 m separation and differnet sites 100's of km apart. Post-hoc analyses showed that this was indeed necessary as distributions were aggregated across scales. |
| Data collection | Field data collection for surveys was by photography, conducted by junior author. Small samples for lab were taken directly with Petri dishes. |
| Timing and spatial scale | Surveys were conducted ad hoc, after rain events on 6 Feb, 7 Mar (2018), 5 Jan , 8-9 Feb, (2019) and 1-3 July, 4 Aug (2021). Scale of a transect was in the order of 50-100 m. Distance among sites was in he order of 100 Km. |
| Data exclusions | No data were excluded |
| Reproducibility | Reproducibility for lab experiments was at least 5 replicates. results for all replicates are presented in the supplementary files |
| Randomization | Not applicable, because all surveys conducted are included in the data analyses |
| Blinding | Blinding was not necessary as the surveys were predetermined in advance of data collection, and all were included. |

Did the study involve field work? ☒ Yes ☐ No

## Field work, collection and transport

| | |
|---|---|
| Field conditions | Samples were taken during/ just after rainfall event so that plaques would become visible. No additional environmental measures were taked during measurements, as the communities are dormant during dry intervening periods. |

| | |
|---|---|
| Location | Sites: lat 32.59194°, long -106.85286; lat 32.50321°, long -106.74097°; lat 32.56348°, long -106.75795°; lat 32.51540°, long -106.74269°; lat 34.33703°, long -106.72910°; lat 32.9913°, long -111.76130°; lat 33.3923°, long -111.35404°;lat 33.57293°, long -111.79713°; lat 33.30089°, long -111.68285° |
| Access & import/export | Sites were either on public lands with no permit needed, on USDA Experimental Range, for which the junior and senior authors have permission to work and sample through the Jornada NSF- LTER (Garcia-Pichel, co-PI), or in ASU property managed by Clearway Energy Group from whom we obtained permission and gate access codes |
| Disturbance | Disturbance was minimal and restricted to foot traffic to sampling sites, minimized by linear access. Retrieval sampling was also minimal consisting of a few 10 cm wide by 1 cm deep soil samples per site. Photography caused no disturbance. |

# Reporting for specific materials, systems and methods

We require information from authors about some types of materials, experimental systems and methods used in many studies. Here, indicate whether each material, system or method listed is relevant to your study. If you are not sure if a list item applies to your research, read the appropriate section before selecting a response.

## Materials & experimental systems

| n/a | Involved in the study | |
|---|---|---|
| ☒ | ☐ | Antibodies |
| ☒ | ☐ | Eukaryotic cell lines |
| ☒ | ☐ | Palaeontology and archaeology |
| ☒ | ☐ | Animals and other organisms |
| ☒ | ☐ | Clinical data |
| ☒ | ☐ | Dual use research of concern |

## Methods

| n/a | Involved in the study | |
|---|---|---|
| ☒ | ☐ | ChIP-seq |
| ☒ | ☐ | Flow cytometry |
| ☒ | ☐ | MRI-based neuroimaging |
